# Coupled Double Closed-Loop Control for an MEMS Resonant Accelerometer

**DOI:** 10.3390/mi13101612

**Published:** 2022-09-27

**Authors:** Heng Liu, Jiale Wu, Yu Zhang

**Affiliations:** School of Electronic & Information Engineering, Nanjing University of Information &Technology, Nanjing 210044, China

**Keywords:** resonant accelerometer, constant amplitude, frequency tracking, loop coupling, averaging method

## Abstract

There is mutual coupling between amplitude control and frequency tracking control in the closed-loop control of micromechanical resonant sensors, which restricts sensor performance. This paper introduces the principle of an in-plane vibration micromechanical resonant accelerometer with electrostatic stiffness. The characteristic parameters of the microaccelerometer were obtained through computer-aided dimension measurement and an open-loop frequency sweep test of the fabricated microstructure. An accurate numerical model was established based on the accelerometer’s dynamic principle and characteristic parameters. We established the double closed-loop driving analysis model of amplitude automatic gain control and resonant frequency phase-locked tracking. We used the averaging method to analyze the steady-state equilibrium point and the stable condition. We concluded that the integral coefficient can improve the startup overshoot when the amplitude automatic gain control loop satisfies the stability condition. Under the constraint of frequency tracking, the sizeable coefficient of the integrator can improve the system instability of the amplitude control loop. The theoretical analysis and simulation were helpful in the design and debugging of the system circuit.

## 1. Introduction

Micromechanical resonant sensors have the advantages of small volumes, low power consumption, and mass production. They are also characterized by their output frequency and have outstanding anti-interference performance. They are widely used in measuring mass, acceleration, pressure, and so on [1,2,3]. Micromechanical resonant sensors must satisfy the whole system phase and amplitude balance condition [4,5]. This requires the closed-loop system to ensure the constancy of the oscillation amplitude of the resonator and its frequency tuning to resonance [6,7]. Three problems need to be discussed and studied in closed-loop control for micromechanical resonant sensors. First, the mathematical modeling of the dynamics of micromechanical resonators is of significant theoretical and applied interest. The core of the micromechanical resonant sensor is the resonator. In system dynamics analysis, the microresonator is equivalent to a second-order linear system [8]. However, it is reported that the microresonator is no longer equivalent to a second-order system under large amplitude vibration and behaves as a nonlinear higher-order oscillator [2,9,10]. The numerical modeling of the microresonator is not scientific enough and does not combine with the actual manufacturing size [11,12]. Take the micromechanical resonant accelerometer as an example. The resonant frequency is more significant than 15 kHz [13], and the quality factor of the vacuum package is more prominent than 500 [14]. Establishing a numerical model based on the actual manufacturing microstructure and packaging test results is more valuable.

Second, dual closed-loop control is necessary to ensure a constant vibration amplitude and resonant frequency tracking. As a rule, constant vibration amplitude is solved using automatic gain control (AGC) technology [15,16]. The electrostatic driving force is related to the AC driving voltage and the DC driving voltage. Automatic gain control also includes the automatic gain control of the AC voltage amplitude (AGC-AC) and the automatic gain control of the DC voltage (AGC-DC). The former depends on a more nonlinear integrated chip circuit, so it is common to use the automatic gain control of DC voltage at present. When the quality factor is relatively significant (Q > 500), the resonator has outstanding frequency selection characteristics and can be driven by self-excitation [17,18,19]. However, the interface circuit generates a phase shift, so the self-excited drive requires a phase compensation circuit [17,18,19]. The phase shift is related to the excitation frequency, and it is not easy to achieve an accurate phase balance of different sensors. Many designs use a phase-locked loop (PLL) to maintain the phase balance [20,21,22]. Both digital and analog phase-locked loops can realize effective frequency tracking [20,21,22]. However, the auto gain control loop and frequency tracking loop are coupled, and the stability conditions of the whole system are also restricted. There has been no previous coupling analysis and simulation verification.

Third, when the amplitude of the micromechanical resonator is large, the microstructure vibrates unsteadily in the nonlinear region [3,23,24]. Maintaining the microstructure’s stable resonance is a problem to be solved by the closed-loop measurement and control circuit. In many works, the nonlinear dynamics and bifurcations of the operating modes of the PLL circuit and AGC circuit are studied [25,26]. There are also known works devoted to studying the nonlinear dynamics of micromechanical resonators in control loops that implement the amplitude and phase stabilization of oscillations. The relevance of the latter direction is especially significant in the context of the tasks of designing architectures and ensuring stable dynamic modes of operation of high-quality sensitive elements of micromechanical inertial navigation systems, where the stability of reaching the required amplitudes of stationary oscillations in the presence of inevitable mechanical and other nonlinearities affects the accuracy of the sensor.

Micromechanical resonant accelerometers have the advantage of high-resolution measurement and are widely used in measuring slight acceleration and displacement [1]. The principle of a silicon-based electrostatic-stiffness-type in-plane resonant accelerometer is introduced in this paper, and the fabrication is realized by inductive coupled plasma (ICP) deep etching and anodic bonding technology. The geometric dimensions of the accelerometer were obtained by computer-vision-aided testing. The resonant frequency and quality factor were obtained by the open-loop frequency sweep test after the accelerometer was vacuum-packaged. The nonlinear phenomenon of the resonant amplitude and frequency coupling of the microstructure beam were observed. Based on the above tests, an accurate numerical simulation model of a micromechanical accelerometer was established. The numerical analysis model of the double closed-loop drive circuit with automatic gain control and phase-locked control was established. The stability condition and steady-state behavior of the accelerometer under the condition of the cubic stiffness coefficient were analyzed by the averaging method. The independent decoupling analysis method was adopted for the coupling of dual loops, and the numerical simulation was used to experiment. The purpose was to solve the nonlinear problem of the system. The stability region of the system was analyzed and estimated, and the simulation and experiment were carried out to improve the stability of the accelerometer.

## 2. MEMS Resonant Accelerometer and Open-Loop Experiment

**Figure 1:**
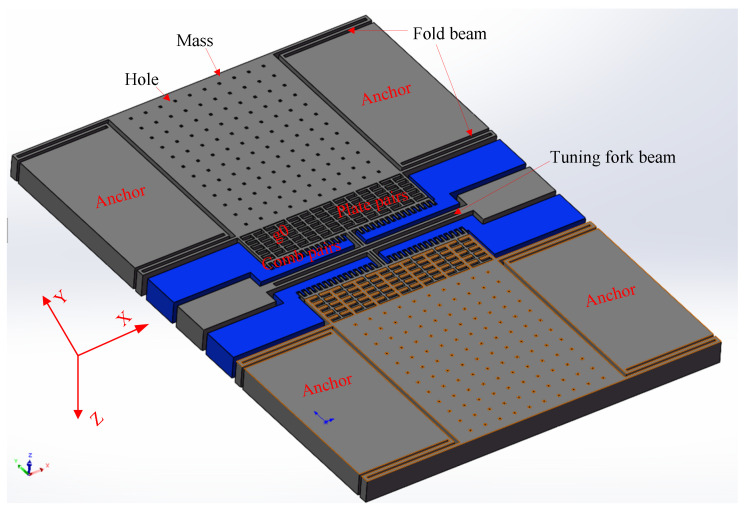
Structural diagram of an accelerometer.

The structure layer of the accelerometer is divided into two identical single-beam resonant accelerometers at the middle symmetry point in the *Y*-axis direction, as shown in Figure 1. Tasking the upper part as an example, the structure layer includes a sensitive proof mass with some damping holes, four single-stage folded beams supporting the suspended proof mass, a detection plate capacitor pair, two fixed-drive comb capacitor pairs, a tuning fork resonant beam, and some fixed anchors (two anchors at the detection end, two anchors for the driving comb, and two anchors for the double-end tuning fork). The *Y*-axis direction is the drive and detection direction.

**Figure 2:**
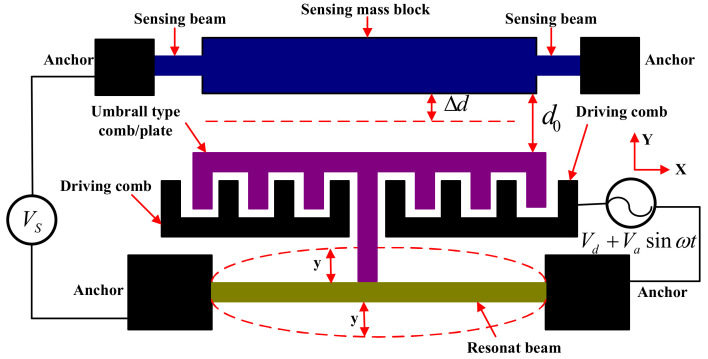
Equivalent diagram of an accelerometer.

An equivalent diagram of an accelerometer is shown in Figure 2. Folded beams and proof mass connect with the detection voltage, $V_{s}$, *V.* Both the driving combs link with driving voltage, $V_{d}+V_{a}\sin\omega t$, *V*. The tuning fork beam potential is 0. The dynamic equation of the resonant beam is [8]:

(1) $$m\ddot{y}+\xi \dot{y}+k_{m}y=F_{d}+F_{e}$$

In Equation (1), y is the modal displacement of the tuning fork beam, *m*; $F_{d}$ is the electrostatic driving force, *N*; $k_{m}$ is the effective mechanical stiffness of the tuning fork beam, *N/m*; *m* is the equivalent mass, *kg*; and the damping coefficient is ξ. The electrostatic force of the detection plate capacitance acting on the resonant beam is $F_{e}$, *N/m*. The total capacitance of the detection plate is:

(2) $$C_{s}=\frac{\varepsilon Nhl}{d_{0}-y_{1}}=\frac{\varepsilon S}{d_{0}-y_{1}}$$

In Equation (2), N represents the pairs of the parallel plate capacitance; $d_{0}$ is the initial distance between two plates of the detection capacitor, *m*; $y_{1}$ is the displacement of the folded beam in the *Y*-axis direction, *m*; ε is the dielectric constant; h is the overlapping thickness of the capacitor plates along the *Z*-axis direction, *m*; l is the overlap length of a single capacitor along the *Y*-axis direction, *m*; and S=Nhl is the equivalent area of the two plates of the detection capacitor, *m*^2^.

The electrostatic force, $F_{e}$, is:

(3) $$F_{e}=\sum \frac{1}{2}\frac{\partial C_{s}}{\partial y_{1}}V_{s}^{2}=\frac{N\varepsilon lhV_{s}^{2}}{2}\left(\frac{1}{d_{0}^{2}}+\frac{2y_{1}}{d_{0}^{3}}+O\left(y_{1}^{2}\right)\right)$$

The total capacitance of the drive comb, $C_{d}$, *F*, is:

(4) $$C_{d}=\frac{N_{0}\varepsilon h\left(l_{0}+y\right)}{d_{1}}$$

In Equation (4), $N_{0}$ represents the pairs of the capacitance of the driving comb; $l_{0}$ is the overlapping length of the combs along the *Y*-axis direction, *m*; $d_{1}$ is the distance between the comb and the next comb in the *X*-axis direction, *m*; $V_{d}$ is the DC driving voltage, *V*; and $V_{a}\sin\omega t$ is the AC driving voltage. The electrostatic driving force, $F_{d}$, is:

(5) $$F_{d}=\sum \frac{1}{2}\frac{\partial C_{d}}{\partial y}{\left(V_{d}+V_{a}\sin\omega t\right)}^{2}=\frac{1}{2}\frac{N_{0}\varepsilon h}{d_{1}}{\left(V_{d}+V_{a}\sin\omega t\right)}^{2}$$

Substituting Equations (3) and (5) into Equation (1) and ignoring the higher-order terms of the electrostatic force, we obtain:

(6) $$m\ddot{y}+\xi \dot{y}+K_{eff}y=\frac{N_{0}\varepsilon h}{2d_{1}}{\left(V_{d}+V_{a}\sin\omega t\right)}^{2}+\frac{\varepsilon SV_{s}^{2}}{2d_{0}^{2}}$$

In Equation (6), the equivalent stiffness of the resonant beam, $k_{eff}$, is:

(7) $$k_{eff}=k_{m}-\frac{\varepsilon SV_{s}^{2}}{d_{0}{}^{3}}$$

According to Equation (7), when the detection terminal is loaded with voltage, $V_{s}$, the resonant frequency decreases when the equivalent stiffness of the resonant beam decreases, and the reduction is related to $V_{s}$ and $d_{0}$. The resonant accelerometer can be designed by establishing the relationship between the acceleration, a, in the *Y*-axis direction and the initial distance, $d_{0}$.

According to Figure 2, the folded beams and the resonant beam were under an acceleration excitation, which made the solution for the distance, $d_{0}$, more complicated. The equivalent stiffness, $k_{s}$, was much smaller than the modal stiffness, $k_{m}$, and the detection proof mass, $m_{s}$, was much larger than that of the resonant beam, m. Under the action of the electrostatic driving force, the resonant beam performed a high-frequency sinusoidal periodic vibration about the fixed equilibrium position, and the equivalent low-frequency displacement was 0.

When the acceleration in the *Y*-axis direction is 0, the detection capacitance system can be calculated as:

(8) $$\frac{1}{2}\frac{\varepsilon SV_{s}^{2}}{{\left(d_{0}-\Delta d\right)}^{2}}=k_{s}\cdot \Delta d$$

When the acceleration in the *Y*-axis direction is not 0, the following holds true:

(9) $$\frac{1}{2}\frac{\varepsilon SV_{s}^{2}}{{\left(d_{0}-\left(y_{1}+\Delta d\right)\right)}^{2}}=k_{s}\left(y_{1}+\Delta d\right)-m_{s}\cdot a$$

In Equations (8) and (9), Δd and $y_{1}$ are the displacements of the folded beam and the proof mass in the *Y*-axis direction when the acceleration is 0 and not 0, *m*, *m*, respectively. The actual design should consider the pull-in effect of the plate capacitor; that is, the value $d_{0}$ should be as large as possible, but if it is too large, the detection of the output signal becomes difficult. Generally, it satisfies $y_{1}+\Delta d\ll d_{0}$. After expansion with the Taylor series, the relationship between displacement, $y_{1}+\Delta d$, and acceleration, a, can be obtained as follows:

(10) $$y_{1}+\Delta d=\frac{m_{s}\cdot a}{k_{s}-\frac{\varepsilon SV_{s}^{2}}{d_{0}^{3}}}+\frac{\frac{\varepsilon SV_{s}^{2}}{2d_{0}^{2}}}{k_{s}-\frac{\varepsilon SV_{s}^{2}}{d_{0}^{3}}}$$

The resonant frequency, $f_{e}$, of the resonant beam is:

(11) $$f_{e}=\frac{1}{2\pi }\sqrt{\frac{k_{m}-\frac{\varepsilon SV_{s}{}^{2}}{{\left(d_{0}-y_{1}\right)}^{3}{\left(1-\frac{-\Delta d}{d_{0}-y_{1}}\right)}^{3}}}{m}}$$

where

$$\Delta d=\frac{\frac{\varepsilon SV_{s}^{2}}{2d_{0}^{2}}}{k_{s}-\frac{\varepsilon SV_{s}^{2}}{d_{0}^{3}}},y_{1}=\frac{m_{s}\cdot a}{k_{s}-\frac{\varepsilon SV_{s}^{2}}{d_{0}^{3}}}$$

Sensitivity, η, is expressed as:

(12) $$\left|\eta \right|\approx \frac{\delta f_{e}}{\delta a}\approx \frac{3\varepsilon SV_{s}{}^{2}/2\pi }{\sqrt{(k_{m}d_{0}{}^{4}-\varepsilon SV_{s}{}^{2}d_{0})\cdot m}}\frac{m_{s}}{2k_{s}d_{0}{}^{2}-3\varepsilon SV_{s}{}^{2}/d_{0}}$$

In Equation (12), the sensitivity can be improved by adjusting the loading voltage, $V_{s}$, but the sensitivity is nonlinear with $V_{s}$. The greater the stiffness of the folded beam, $k_{s}$, the smaller the sensitivity. It is necessary to configure the parameters reasonably in the structural design. The preceding analysis was conducted under the condition $k_{s}\ll k$; that is, the influence of the tuning fork beam on the resonant frequency by the electrostatic force and inertial force can be ignored.

**Figure 3:**
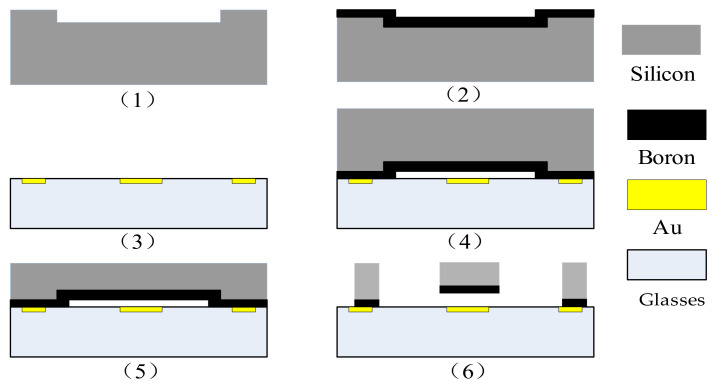
Bulk silicon technological process. (1) clean the monocrystalline silicon wafer and etch the bonding platform; (2) dope with concentrated boron via a diffusion process;(3) sputter Au on the borosil-icate glass; (4) bond the glass and silicon through the anode; (5) dry-etched with excess silicon; (6) etch the back of the silicon wafer.

**Figure 4:**
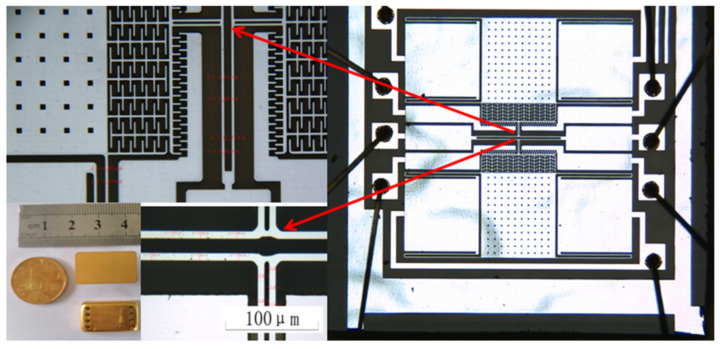
The design of the MEMS accelerometer.

The microaccelerometer uses monocrystalline silicon doped with concentrated boron as the structural layer material, and the bond material is Pyrex7740 glass. An inductively coupled plasma (ICP) deep silicon etching process is used to obtain a much greater etching depth-to-width ratio for the microstructure. Process flow: in step 1, the monocrystalline silicon wafer is cleaned, and the bonding platform is etched; in step 2, monocrystalline silicon is doped with concentrated boron by a diffusion process to increase the conductivity of the structure; in step 3, Au is sputtered on the Pyrex7740 glass as an electrode; in step 4, the glass and silicon are bonded; in step 5, the back of the bonded silicon wafer is drily etched, and the structural layer is thinned; in step 6, the back of the silicon wafer is etched with the deep silicon etching process. The gap between the structure and the substrate is easy to control. The process flow has fewer pollution impurities, only three masks, and photolithography. The process flow is shown in Figure 3, and Figure 4 shows the accelerometer core and the corresponding package. A computer vision method directly marked the length and width dimensions. Table 1 shows the structural parameters that were designed and measured.

**Figure 5:**
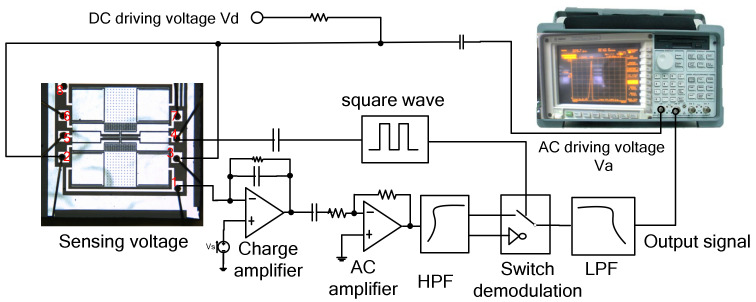
Open-loop parameter measurement circuit. The lower electrode connected to sensing voltage; The lower electrode connected to driving voltage; The electrode connected to the tuning fork beam; The upper electrode connected to driving voltage; The upper electrode connected to sensing voltage.

The quality factor and resonant frequency are essential parameters for the microaccelerometer. In Figure 5, the microaccelerometer has eight electrodes, among which the fourth and fifth electrodes are the resonant beam electrodes, the first electrode is the detection electrode of the lower single beam, and the second and third electrodes are the corresponding driving electrodes; the eighth electrode is the detection electrode of the single beam, and the sixth and seventh electrodes are the corresponding detection electrodes.

**Figure 6:**
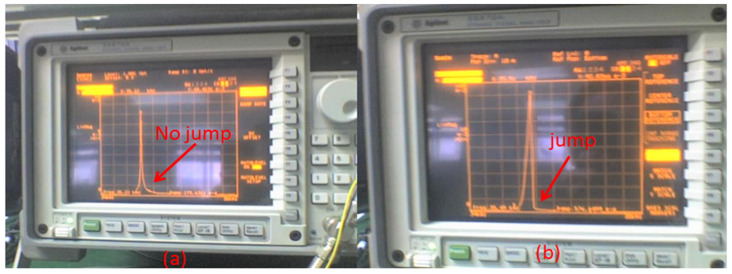
Amplitude–frequency curve of single resonant beam. In the amplitude–frequency curve (**a**) there is no jump; (**b**) there is a jump.

Taking a single-beam accelerometer as the experiment, the AC driving voltage, $V_{a}(t)$, is provided by the first channel of the Agilent35670A dynamic signal analyzer, and the DC driving voltage, $V_{d}$, is provided by the DC regulated power supply. The open-loop test circuit includes the accelerometer core, charge amplifier, isolated DC amplifier, band-pass filter, analog multiplier, low-pass filter, and high-frequency sinusoidal wave generation module. The detection electrode (the first electrode) is connected to the detection voltage, $V_{S}$, through the charge amplifier, and the signal filtered by the low-pass filter is connected to the second channel of the Agilent35670A. To eliminate the same frequency interference of parasitic capacitance, a high-frequency sine wave modulation and analog multiplier demodulation are adopted. The high-frequency sine wave is connected to the resonant beam electrode (the fourth electrode). Figure 6 shows the amplitude–frequency curve obtained by the accelerometer open-loop test when the acceleration is 0, the *X* axis is the frequency of the AC driving voltage, and the *Y* axis is the ratio of voltage magnitude (0.001/div). The test shows that under the condition of a vacuum package (20–30 mTorr), when the frequency sweep range is 33–40 kHz, the frequency sweep is 250 points. When the voltage is 3 V, there is no amplitude, and frequency curve jump is shown in Figure 6a. The corresponding resonance frequency is 35.16 kHz, and the quality factor is 1476. When the voltage is 5 V, the corresponding resonance frequency is 35.746 k Hz, and there is a jump in the amplitude and frequency curve, as shown in Figure 6b. As the voltage increases, the corresponding resonance frequency increases, and the vibration amplitude also increases.

An amplitude jump is observed on the right side of the resonant point. Further experimental analysis shows that the larger the driving voltage is, the amplitude–frequency curve has the characteristics of the duffing equation, and a nonlinear coupling relationship is observed between the vibration amplitude and the resonant frequency. The dynamic characteristics of a single-beam resonant accelerometer have the attributes of the duffing equation [25]:

(13) $$m\ddot{x}+c\dot{x}+k_{m}x+k_{3}x^{3}=F_{d}(t)$$

In Equation (13), c is the damping coefficient, $k_{3}$ is the nonlinear stiffness coefficient, and the value of $k_{3}$ is related to $k_{m}$ and the width of the tuning fork beam [27]. The electrostatic driving force is $F_{d}(t)$. For Equation (13), the normalization can be expressed as:

(14) $$\ddot{x}+\mu \dot{x}+\alpha x+\beta x^{3}=F_{d}(t)/m$$

In Equation (14), μ=c/m, $\alpha =k_{m}/m$, and $\beta =k_{3}/m$. Combined with the microstructure size measurement of computer vision and the open-loop test experiment, the relevant parameters of the accelerometer are shown in Table 2.

**Figure 7:**
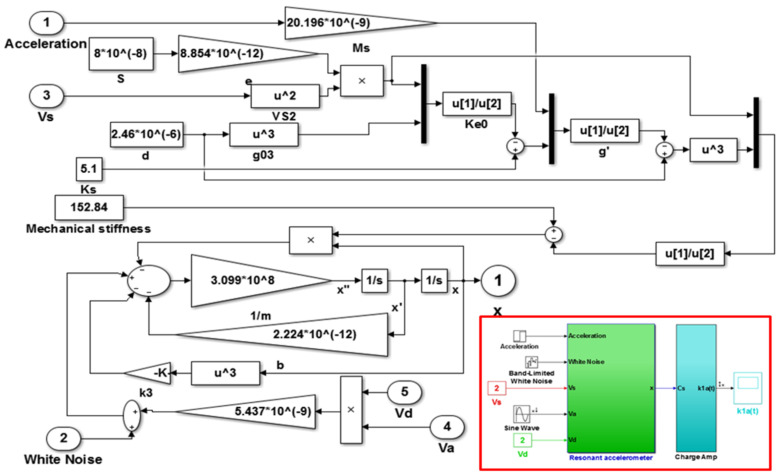
Numerical model of single-beam resonant accelerometer.

According to Table 2 and Equations (1)–(13), the numerical simulation model of the accelerometer based on Matlab/Simulink was established, as shown in Figure 7. The resonance frequency of the microstructure obtained by the open-loop test is 35.16 kHz, and the theoretical calculation of the resonance frequency is 34.546 kHz. Two factors mainly cause the gap between the two. First, the theoretical analysis is based on an ideal model, ignoring the influence of some connecting beams. Second, there is human error in the manual calibration measurement.

## 3. Closed-Loop Drive Control of MEMS Resonant Accelerometer

**Figure 8:**
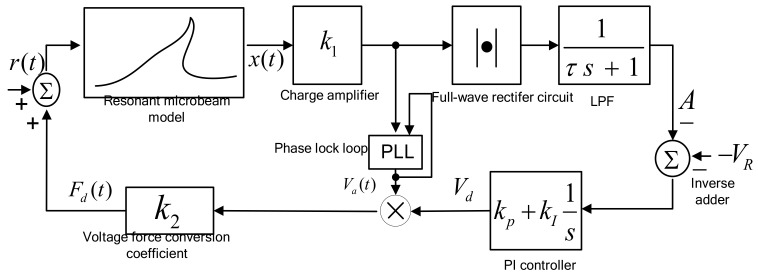
Block diagram of resonant microaccelerometer.

The double closed-loop control circuit block diagram is shown in Figure 8. The hybrid control of the amplitude and frequency includes two loops: a constant amplitude control loop and a resonant frequency tracking control loop, where x(t) is the displacement of the resonant beam, *m*; $k_{1}$ is the displacement detection amplification gain; τ is the time constant of the first-order low-pass filter, *S*; A is the detection amplitude of the displacement signal, *V;* $V_{R}$ is the DC reference voltage, *V*; and the negative sign is because the actual circuit uses an inverse adder to complete the difference calculation. $k_{p}$ and $k_{I}$ are the proportional and integral coefficients, respectively; $k_{2}$ is the conversion coefficient of the voltage and electrostatic force, *F/V^2^*, and r(t) is the equivalent electrostatic force generated by electric noise, *N*, which is far less than the steady-state electrostatic driving force, $F_{d}(t)$, when stable.

The two loops are coupled to each other and assumed to be in a stable state during the analysis. Under the condition that the output voltage amplitude of the voltage-controlled oscillator (VCO) is constant, the range of DC driving voltage is determined according to the detection ability of the interface circuit, and the DC voltage of the frequency tracking loop is determined. Finally, other parameters of the amplitude control loop are determined. In the double closed-loop design process, the frequency tracking loop is first analyzed, and the driving DC voltage is assumed to be constant, so the stability condition of the phase-locked loop is solved, and the critical integral controller coefficient is determined. In the amplitude control loop, the DC reference voltage and other electrical parameters are adjusted to meet the stability conditions of the frequency tracking loop.

### 3.1. Frequency Tracking Control Based on Phase-Locked Technology

**Figure 9:**
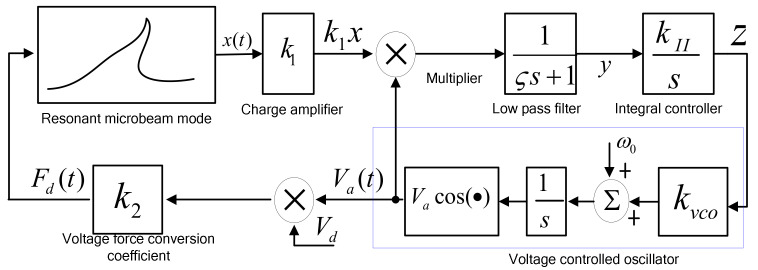
Frequency tracking control of resonant microaccelerometer. This rectangle represents voltage controlled oscillator (VCO).

The frequency tracking control loop includes an accelerometer core, a charge amplifier, an analog multiplier, a low-pass filter, an integral controller, and a VCO. In the initial state, the DC driving voltage is constant, the AC voltage is generated by the VCO, and the initial frequency is set according to the design resonant frequency. When the phase error between the detected displacement signal and the driving AC voltage is not $90^{{}^{\circ}}$, the integral controller accumulates the phase error to adjust the input voltage of the VCO [22]. When the whole system is stable, the output frequency of the VCO is consistent with the resonant frequency of the resonant beam, and the analysis model is shown in Figure 9.

In Figure 9, ς is the time constant of the first-order low-pass filter, *S*; $k_{II}$ is the integral coefficient, and it is affected by controller design; y is the input voltage of the integrator, *V*; z is the control voltage of the VCO, *V*; $k_{vco}$ is the conversion coefficient of the VCO, π/V; and $V_{a}$ and $\omega_{0}$ are the output voltage amplitude and initial oscillation frequency of the VCO, respectively. The quality factor of the accelerometer is more than 500, and the electrostatic force is simplified as:

(15) $$F_{d}(t)=k_{2}\cdot V_{d}\cdot V_{a}\cos(\omega t+\phi )=F\cos(\theta (t)).$$

In Equation (15), $k_{2}$ is the voltage–electrostatic force conversion coefficient, *F/V^2^*, which is related to the driving comb capacitance parameters, ω is the frequency of the VCO, φ is the initial phase, θ(t) is the real-time phase, F is the amplitude of the driving force, the frequency tracking loop driving voltage amplitude is constant, and F is also constant. For voltage-controlled oscillators:

(16) $$\dot{\theta }=\omega_{0}+k_{vco}z.$$

For the integral controller of the phase angle difference:

(17) $$\dot{z}=k_{II}y.$$

For the analog multiplier phase discriminators:

(18) $$\dot{y}=\varsigma (k_{1}\cdot x(t)\cdot V_{a}\cos(\theta (t))-y).$$

According to the modeling of the above modules, the system dynamic equation is:

(19) $$\left\{\begin{array}{l} (m\ddot{x}+c\dot{x}+kx+k_{3}x^{3})=F\cos\theta =I\cos\theta \\ \dot{\theta }=\omega_{0}+k_{vco}\cdot z\\ \dot{z}=k_{II}\cdot y\\ \dot{y}=\varsigma (k_{1}\cdot x\cdot V_{a}\cos\theta -y) \end{array}\right..$$

It is assumed that the displacement of the resonant beam is x(t):

(20) x(t)=a(t)cos(θ(t)+ϕ(t)),

where a(t) and ϕ(t) are the amplitude and phase of the vibration displacement, respectively. Under the condition of stability, both are slowly varying parameters. The vibration velocity of the resonant beam can be obtained by differentiating the displacement:

(21) $$\dot{x}(t)=-a(t)\dot{\theta }\sin(\theta +\phi )+\dot{a}(t)\cos(\theta +\phi )-a(t)\dot{\phi }\sin(\theta +\phi ).$$

As the amplitude and phase are slowly changing parameters:

(22) $$\dot{a}(t)\cos(\theta +\phi )-a(t)\dot{\phi }\sin(\theta +\phi )=0.$$

Thus, the vibration acceleration is:

(23) $$\ddot{x}(t)=-\dot{a}(t)\dot{\theta }\sin(\theta +\phi )-a(t)\dot{\theta }(\dot{\theta }+\dot{\phi })\cos(\theta +\phi ).$$

Substituting $\ddot{x}(t)$, $\dot{x}(t)$, and x(t) into Equation (19) and combining it with Equation (22), the averaging method can be used to simplify:

(24) $$\dot{a}(t)=-\frac{1}{2\omega }[I\sin(\phi )+a(t)(\mu \omega +k_{II}k_{vco}y)],$$

(25) $$\dot{\phi }(t)=-\frac{1}{2\omega }[\frac{I}{a(t)}\cos(\phi )+[\omega^{2}-(\alpha +3/4\beta a^{2}(t))]],$$

(26) $$\dot{\omega }=k_{II}\cdot k_{vco}\cdot y,$$

(27) $$\dot{y}=\varsigma (\frac{1}{2}k_{1}\cdot \bar{a}(t)\cdot V_{a}\cos\phi -y).$$

For the solution of the equilibrium point, let the derivative of each parameter with respect to time *t* be 0. Then:

$$\bar{a}=\frac{I}{\mu \bar{\omega }},\bar{\phi }=-\frac{\pi }{2},\bar{y}=0,$$

$$\bar{\omega }=\sqrt{\frac{1}{2}(\alpha +\sqrt{\alpha^{2}+3\beta I^{2}/\mu^{2}})}.$$

According to the expressions of $\bar{a}$ and $\bar{\omega }$, the amplitude of the steady state is related not only to I but also to μ and β. The larger μ and β are, the smaller their amplitude is. To analyze the stability of the loop, the equation was linearized at the equilibrium point, and the Jacobian matrix was obtained:

(28) $$\left[\begin{array}{l} \dot{a}(t)\\ \dot{\phi }(t)\\ \dot{\omega }(t)\\ \dot{y}(t) \end{array}\right]=\left\lfloor \begin{array}{cccc} -\frac{\mu }{2} & 0 & -\frac{I}{2{\bar{\omega }}^{2}} & -\frac{\bar{a}k_{vco}k_{II}}{2\bar{\omega }}\\ \frac{3\bar{a}\beta }{4\bar{\omega }} & -\frac{\mu }{2} & -1 & 0\\ 0 & 0 & 0 & k_{II}k_{vco}\\ 0 & \frac{\varsigma k_{1}\bar{a}}{2} & 0 & -\varsigma \end{array}\right\rfloor \left[\begin{array}{l} a(t)\\ \phi (t)\\ \omega (t)\\ y(t) \end{array}\right].$$

The characteristic root equation that corresponds to the system is:

(29) $$n_{0}\lambda^{4}+n_{1}\lambda^{3}+n_{2}\lambda^{2}+n_{3}\lambda +n_{4}=0.$$

In Equation (29), λ is the eigenvalue. $n_{0},n_{1},n_{2},n_{3},n_{4}$ are the coefficients of the corresponding items, which can be expressed as:

$$M=k_{1}\cdot k_{II}\cdot k_{vco}\cdot \varsigma >0$$

$$n_{0}=1>0$$

$$n_{1}=\mu +\varsigma >0$$

$$n_{2}=\mu (\frac{\mu }{4}+\varsigma )>0$$

$$n_{3}=\frac{\mu^{2}\varsigma }{4}+\frac{M\cdot I}{2\mu \cdot \bar{\omega }}>0$$

$$n_{4}=\frac{M\cdot I}{4\bar{\omega }}>0$$

According to the Routh criterion, to make the system stable, it is necessary to satisfy:

(30) $$(n_{1}\cdot n_{2}-n_{0}\cdot n_{3})\cdot n_{3}-n_{1}{}^{2}\cdot n_{4}>0.$$

By solving Equation (30), we can obtain:

(31) $$k_{II}<\frac{\mu^{2}\bar{\omega }}{2I\cdot k_{1}\cdot k_{vco}}(\varsigma +\mu ).$$

The integral coefficient, $k_{II}$, in the controller must meet Equation (31). The critical integral coefficient, $k_{II}$, is related to the DC driving voltage, $V_{d}$, which is coupled with the amplitude control loop. When the driving voltage increases, I increases and the $k_{II}$ critical value decreases. The same changes apply to $k_{vco}$ and $k_{1}$.When β is greater than 0, that is, under the condition of stiffness “hardening,” the resonant frequency and the critical integral coefficient increase. Under the same conditions, the stability of the system does not change when β increases. The smaller the integral coefficient, the smaller the absolute value of the corresponding eigenvalue, the slower the dynamic response of the system, and the longer the stability time of frequency tracking.

### 3.2. Amplitude Automatic Gain Control

The amplitude automatic gain control and phase tracking control are coupled. In the analysis, the DC amplitude automatic gain control (AGC-DC) based on vibration displacement detection was mainly analyzed, and the frequency tracking loop was assumed to be frequency-locking. The dynamic equation of the amplitude control loop in Figure 8 is expressed as:

(32) $$\left\{\begin{array}{l} m(\ddot{x}+\omega_{n}/Q\dot{x}+\omega_{n}{}^{2}x)+k_{3}x^{3}=F_{d}(t)\\ \dot{A}=\frac{1}{\tau }(\left|k_{1}x\right|-A)\\ {\dot{V}}_{d}=k_{p}({\dot{V}}_{R}-\dot{A})+k_{I}(V_{R}-A) \end{array}\right.,$$

where $\omega_{n}=\sqrt{k_{m}/m}$, which is the natural frequency of the resonant beam, and $Q=\sqrt{k_{m}\cdot m}/c$, which is the quality factor because the quality factor, Q, is large, which simplifies to $\frac{1}{2}k_{3}\cdot a^{2}(t)\cdot x$ for $k_{3}\cdot x^{3}$. By substituting Equations (20), (21) and (23) into Equation (32) and combining with Equation (22), the following equations are obtained:

(33) $$\dot{\phi }(t)=\frac{k_{3}\cdot a^{2}(t)\cos^{2}(\theta +\phi )}{2m\omega_{{}_{n}}}+[\frac{k_{2}\cdot V_{d}\cdot V_{a}}{a(t)m\omega_{n}}-\frac{\omega_{n}}{Q}]\cdot \sin(\theta +\phi )\cos(\theta +\phi )$$

(34) $$\dot{a}(t)=\frac{k_{3}\cdot a^{3}(t)\sin(\theta +\phi )\cos(\theta +\phi )}{2m\omega_{{}_{n}}}+[\frac{k_{2}\cdot V_{d}\cdot V_{a}}{m\omega_{n}}-\frac{\omega_{n}}{Q}a(t)]\cdot \sin^{2}(\theta +\phi )$$

(35) $$\dot{A}=\frac{1}{\tau }(\frac{2}{\pi }k_{1}a(t)-A)$$

(36) $${\dot{V}}_{d}=-k_{p}\dot{A}+k_{I}(V_{R}-A)$$

When $k_{3}$ is small and Q is large, the system still has weak nonlinear characteristics. By using the characteristics of the slow time-varying system, the stability of the system constituted by Equation (32) was analyzed, and the equilibrium point was solved as follows:

(37) $$\left\{\begin{array}{l} \bar{a}(t)=\frac{\pi V_{R}}{2k_{1}}\\ \bar{A}=V_{R}\\ {\bar{V}}_{d}=\frac{\pi m\omega_{n}{}^{2}V_{R}}{2k_{1}k_{2}Q} \end{array}\right..$$

Equation (32) is approximately linearized near the equilibrium point:

(38) $$\left[\begin{array}{l} \dot{a}(t)\\ \dot{A}(t)\\ {\dot{V}}_{d}(t) \end{array}\right]=\left[\begin{array}{l} \begin{array}{ccc} -\frac{\omega_{n}}{2Q} & \begin{array}{ccc} & 0 & \end{array} & \frac{k_{1}k_{2}}{2m\omega_{n}} \end{array}\\ \begin{array}{ccc} \frac{2k_{1}}{\tau \pi } & \begin{array}{ccc} & -\frac{1}{\tau } & \end{array} & 0 \end{array}\\ \begin{array}{ccc} -\frac{2k_{1}k_{p}}{\tau \pi } & \frac{k_{p}-\tau k_{III}}{\tau } & 0 \end{array} \end{array}\right]\left[\begin{array}{l} a(t)\\ A(t)\\ V_{d}(t) \end{array}\right].$$

The corresponding characteristic equation is:

(39) $$\lambda^{3}+(\frac{\omega_{n}}{2Q}+\frac{1}{\tau })\lambda^{2}+(\frac{\omega_{n}}{2Q}\frac{1}{\tau }+\frac{k}{2m\omega_{n}}\frac{2k_{1}k_{p}}{\tau })\lambda +\frac{k_{1}k_{2}}{2m\omega_{n}}\frac{2k_{1}}{\tau \pi }(\frac{2k_{p}}{\tau }-k_{I})=0.$$

According to the Routh criterion, the corresponding system stability should meet the following conditions:

(40) $$\Delta =\frac{k_{1}k_{2}}{2m\omega_{n}}\frac{2k_{1}}{\tau \pi }(\frac{2k_{p}}{\tau }-k_{I})>0.$$

That is, the following condition needs to be met:

(41) $$k_{I}<\frac{2k_{p}}{\tau }.$$

The pole distribution of the system is changed by different $k_{p}$, $k_{I}$, and τ values so that the system has different dynamic performances. The analysis of the equilibrium point suggests that the steady state value of the vibration amplitude, a(t), is independent of the quality factor and frequency, the amplitude keeps the original value under the condition where the acceleration changes, and the control method can achieve a constant amplitude vibration.

## 4. Simulation Verification

**Figure 10:**
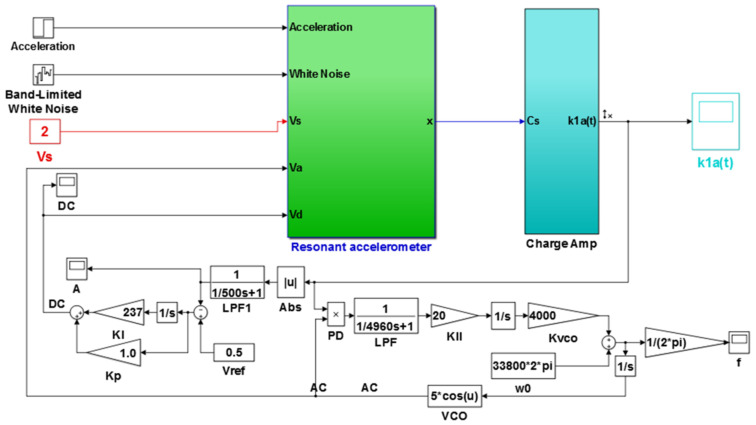
Simulation model of double closed-loop control for a resonant accelerometer.

**Figure 11:**
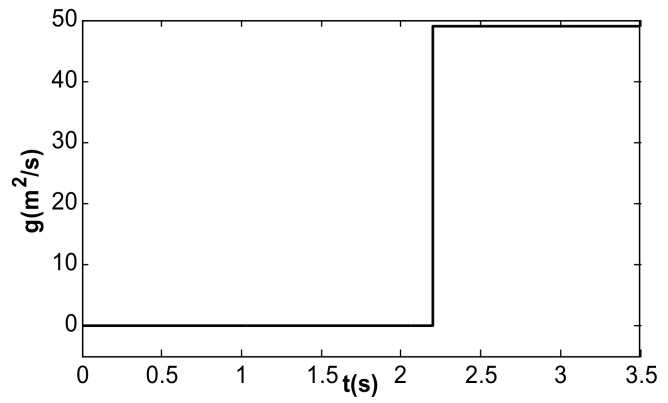
Loaded acceleration signal.

The numerical simulation model of the resonant accelerometer based on Matlab/Simulink (MathWorks Ink. Natick, Massachusetts, U.S.) is shown in Figure 10. When the simulation time was 2.2 s, the acceleration changed from 0 to 49 m^2^/s, as shown in Figure 11.

**Figure 12:**
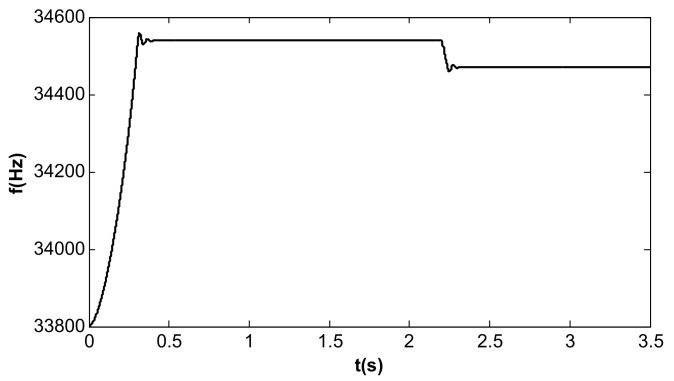
Frequency tracking control under single closed loop ($k_{II}$ = 2).

**Figure 13:**
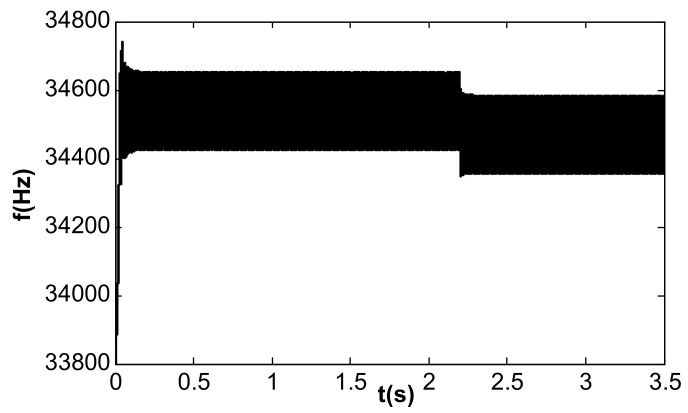
Jitter of the frequency tracking under single closed loop ($k_{II}$ = 60).

In the simulation of the frequency tracking loop, the $V_{R}$ of the DC reference voltage was 0.5 V, and the steady-state DC driving voltage, $V_{d}$, was calculated to be 0.067 V according to Table 2 and Equation (37). According to Equation (31), the critical value of $k_{II}$ in the phase-locked loop was 39.6. When $k_{II}$ was 2, smaller than the critical value, the frequency tracking took a long time to reach stability without frequency jitter, as shown in Figure 12. When $k_{II}$ was 60, larger than the critical value, the time for frequency tracking to reach stability was short, but obvious jitter appeared, as shown in Figure 13.

**Figure 14:**
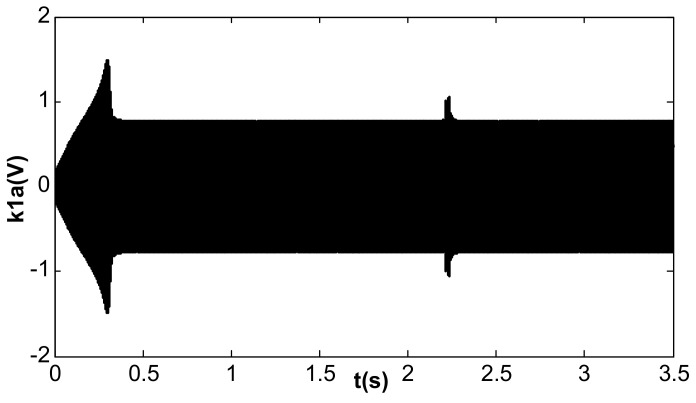
Vibration amplitude under double closed loop ($k_{I}$ = 100, $k_{II}$ = 2).

**Figure 15:**
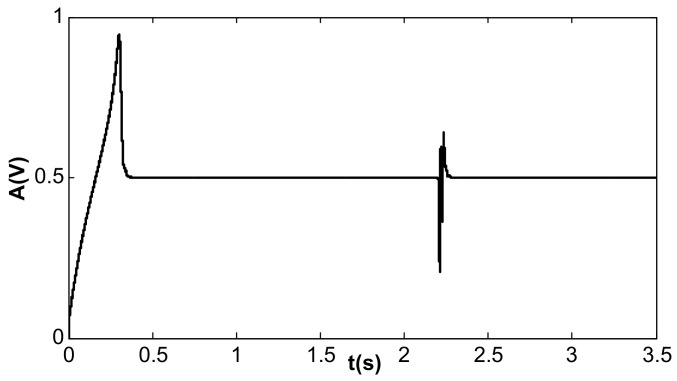
Detection voltage under double closed loop ($k_{I}$ = 100, $k_{II}$ = 2).

**Figure 16:**
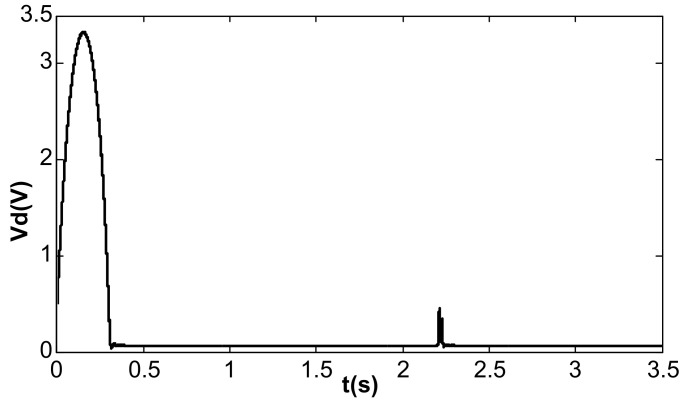
DC driving voltage under double closed loop ($k_{I}$ = 100, $k_{II}$ = 2).

When the value of $k_{II}$ was 2 and the amplitude closed-loop control was added, the $k_{p}$, $k_{I}$, and the filter time constant, τ, became 1.0, 100, and 0.002, which met Equation (41). The DC reference voltage, $V_{R}$, was set as 0.5 V, and the excitation acceleration remained unchanged. According to Equation (26), the amplified steady-state vibration amplitude, k1 a ($k_{1}\cdot \bar{a}$), was 0.785 V, A was 0.5 V, and $V_{d}$ was 0.067 V. Figure 14, Figure 15 and Figure 16 show the simulation results. The simulation steady-state value was completely consistent with the theoretical calculation.

**Figure 17:**
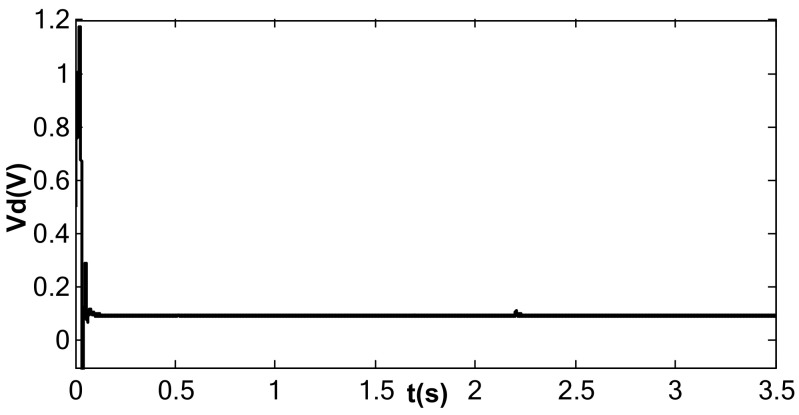
Amplitude fluctuation under double closed loop ($k_{I}$ = 100, $k_{II}$ = 60).

Under the condition that the parameters of the amplitude control loop remain unchanged, jitter of frequency tracking occurred when $k_{II}$ was changed to 60, which affected the DC driving voltage, $V_{d}$, in the amplitude loop. The average value of $V_{d}$ changed to 0.95, thereby showing the characteristics of sinusoidal jitter, as shown in Figure 17.

**Figure 18:**
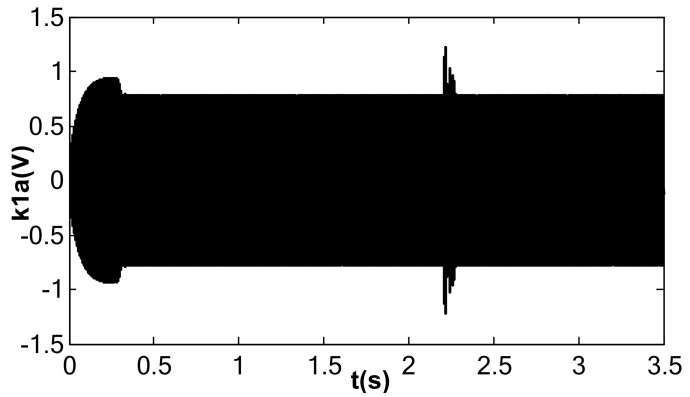
Vibration amplitude when frequency meets requirements ($k_{I}$ = 236, $k_{II}$ = 2).

Figure 18 shows the amplifying amplitude signal when $k_{II}$ was changed to 2 and $k_{I}$ was changed from 100 to 236. Compared with Figure 14, the amplitude overshoot after starting was suppressed better.

**Figure 19:**
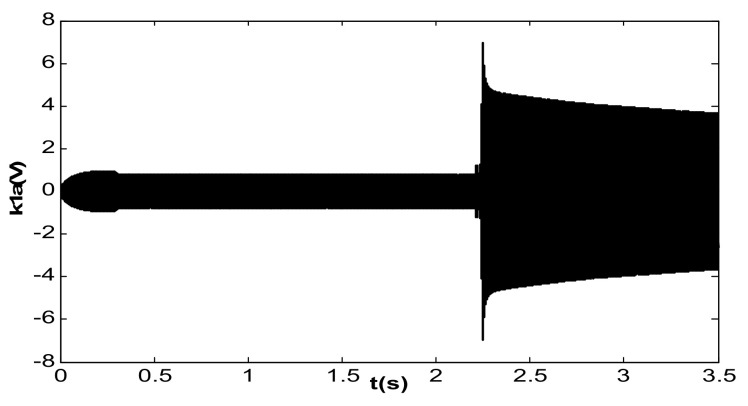
Vibration amplitude when the frequency met the requirements ($k_{I}$ = 240, $k_{II}$ = 2).

**Figure 20:**
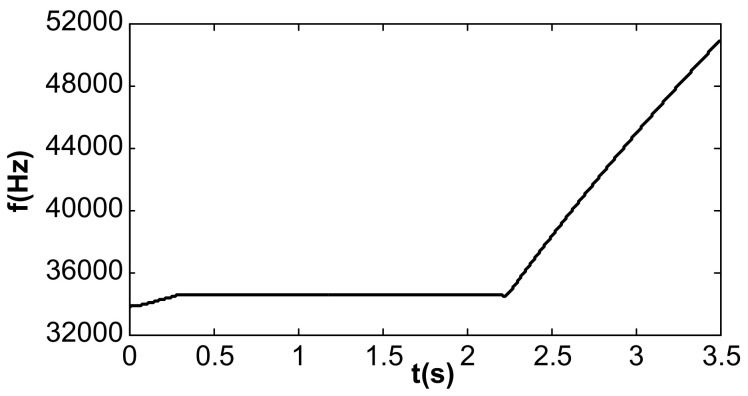
Frequency tracking under double closed loop ($k_{I}$ = 240, $k_{II}$ = 2).

**Figure 21:**
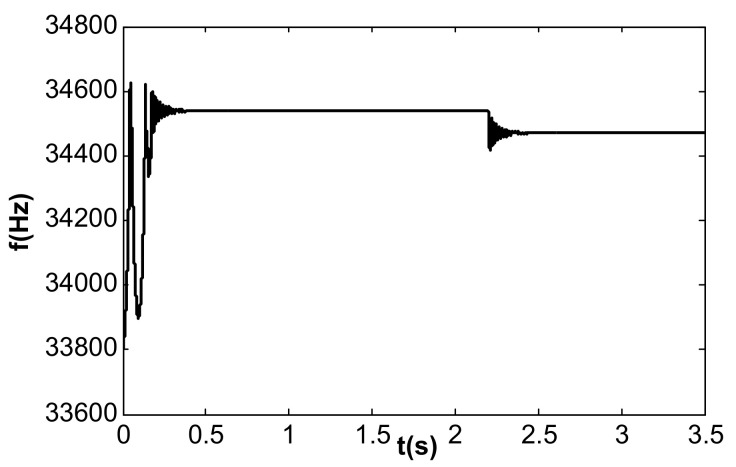
Frequency tracking under double closed loop ($k_{I}$ = 240, $k_{II}$ = 20).

**Figure 22:**
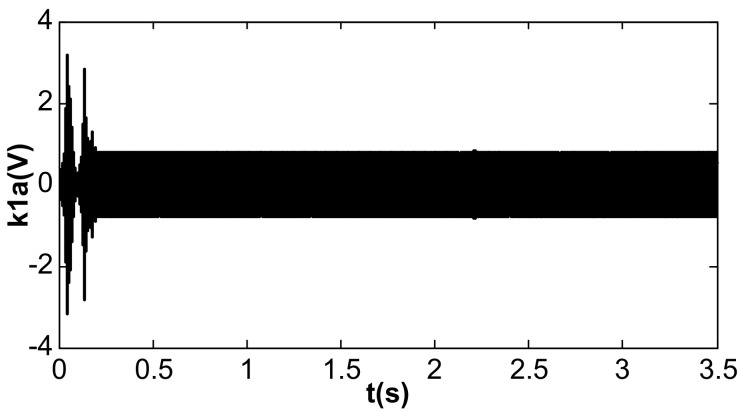
Vibration amplitude of double closed loop ($k_{I}$ = 240, $k_{II}$ = 20).

The value of $k_{I}$ was further changed to 240, and the value of $k_{II}$ remained unchanged. Under this condition, double closed loop control could achieve stable track resonant frequency and a constant amplitude of the accelerometer before acceleration jumping. After acceleration jumping, the decrease in the resonance frequency caused the DC drive voltage to change to a negative value, the absolute value increased continuously, and the vibration amplitude increased continuously, as shown in Figure 19. At the same time, the frequency tracking loop diverged, as shown in Figure 20. The frequency increased continuously, and the system showed instability. When $k_{I}$ was kept unchanged and $k_{II}$ was increased to 20, the system could be stabilized again. However, the amplitude control loop resulted in a large frequency jump of the frequency tracking loop in the start-up stage, as shown in Figure 21, which also led to vibration amplitude jitter, as shown in Figure 22. After the action of the double closed loop, the system asymptotically recovered to stability.

**Figure 23:**
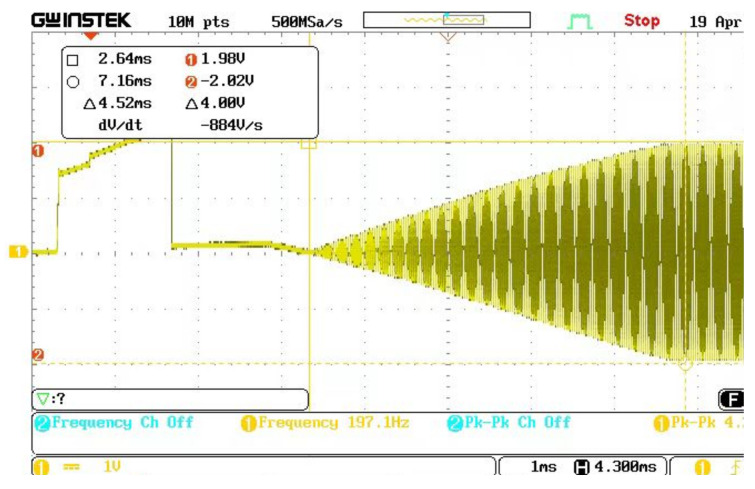
Vibration amplitude of double closed loop.

**Figure 24:**
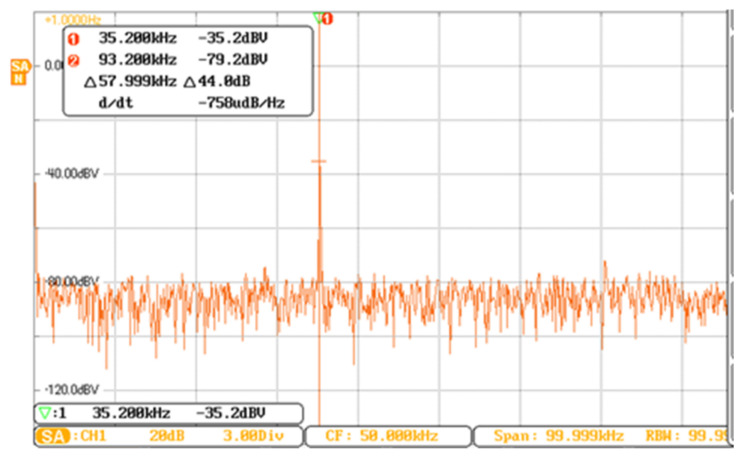
Spectrum of the vibration amplitude signal.

Figure 23 is a vibration output signal of a resonant beam controlled by a double closed loop, and the vibration amplitude is constant. The signal frequency spectrum in Figure 23 was analyzed after linear transformation. The frequency of the resonance signal was 35.20 kHz, and the resonance frequency was consistent with the open-loop frequency sweep test experiment shown in Figure 24. When we adjusted the DC reference voltage, the vibration amplitude of the microstructure changed accordingly. The system could not start vibration when the DC reference voltage was too small. Changing the time constant of the filter could adjust the starting time of the system. The experimental debugging conclusion is consistent with the theoretical analysis.

## 5. Conclusions

Due to the nonlinear coupling problem between the micromechanical resonant accelerometer’s vibration amplitude and the resonance frequency, the numerical model of dual closed-loop control was established. The accelerometer’s stability condition and steady-state equilibrium point were obtained using the averaging method. The following conclusions are brought to guide circuit design and debugging:

First, the amplitude control loop and frequency tracking loop are coupled. When the DC reference voltage, $V_{R}$, is constant, the DC driving voltage, $V_{d}$, is calculated according to Equation (37), and the critical integral coefficient, $k_{II}$, of the frequency tracking loop is calculated by combining $V_{d}$ and Equation (31). If $k_{II}$ is too small, the frequency tracking stability time is too long. If $k_{II}$ is too large, jitter will appear in frequency tracking, and the steady-state equilibrium point of the vibration amplitude is related to the frequency.

Second, when the amplitude of the AC driving voltage in the frequency tracking loop is constant for the amplitude control loop, the integral coefficient, $k_{I}$, in the proportional integral controller must satisfy Equation (41); otherwise, the system becomes unstable. Increasing $k_{I}$ can improve the vibration starting overshoot, but Equation (41) is a necessary and insufficient condition for the system’s stability. After adding the PI controller, the steady state value of the vibration amplitude is independent of the resonant frequency and the quality factor.

Third, when the amplitude control loop satisfies Equation (41) in double closed-loop control, the integral coefficient of the frequency tracking loop increases when the critical condition is satisfied, thereby improving the stability of the amplitude control loop; the instability of the amplitude control loop causes great jitter on the frequency tracking loop. In the double closed-loop design, the convergence of the transition process should be considered as well as the steady-state equilibrium point.

## Figures and Tables

**Table 1:** Structural parameters of the accelerometer.

Parameters	Units	Design	Measurement
Length of fold beam	micron	500	472.5
Width of fold beam	micron	8	7.04
Spacing of fold beams	micron	14	16.2
Length of connecting beam	micron	160	148.4
Width of connecting beam	micron	9	7.5
Spacing of connecting beams	micron	4	4.95
Length of drive comb	micron	40	38.5
Width of drive comb	micron	5	4.79
Spacing of drive combs	micron	2	2.5
Pairs of drive combs	pair	19	19
Length of DETF	micron	700	661.6
Width of DETF	micron	8	7.4
Length of detection plate capacitor	micron	50	45.2
Width of detection plate capacitor	micron	6	4.87
Spacing of detection capacitor 1	micron	2	2.46
Spacing of detection capacitor 2	micron	10	10.56
Total pairs of detection capacitors		40	40
Length of proof mass	micron	620	609
Width of proof mass	micron	700	683
Length of damping hole	micron	10	12.41
Width of damping hole	micron	10	12.93
Number of damping holes		110	110
Structure layer thickness	micron	40	40.4

**Table 2:** Accelerometer structural parameters and circuit parameters.

Parameter	Value	Parameter	Value
k	152.84 N/m	$C_{d0}$	0.88 pF
$k_{s}$	5.1 N/m	$k_{3}$	2.093 × 10^12^ N/m^3^
m	3.312 ug	$C_{s}{}_{0}$	0.39 pF
$m_{s}$	20.196 ug	$d_{1}$	2.5 um
$V_{s}$	2 V	Q	1476
s	0.08 mm × mm	d_0_	2.46 um
$k_{1}$	1,000,000	h	40.4 um
$N_{0}$	38	ε	8.854 × 10^−12^

## Data Availability

All data are true and reliable.

## References

[1] Wang S., Wei X., Zhao Y., Jiang Z., Shen Y. (2018). A MEMS resonant accelerometer for low-frequency detection. Sens. Actuators A.

[2] Li L., Liu H., Shao M., Ma C. (2021). A Novel Frequency Stabilization Approach for Mass Detection in Nonlinear Mechanically Coupled Resonant Sensors. Micromachines.

[3] Brunner D., Yoo H., Schitter G. (2021). Linear Modeling and Control of Comb-Actuated Resonant MEMS Mirror with Nonlinear Dynamics. IEEE Trans. Ind. Electron..

[4] Yang B., Wang X., Dai B., Liu X. (2015). A New Z-axis Resonant Micro-Accelerometer Based on Electrostatic Stiffness. Sensors.

[5] Zhu F., Chen J., Guo H., Liu M., Han S. (2020). A Drive Control Method for Silicon Micro-gyroscopes. Sens. Mater..

[6] Chen F., Yuan Z., Chang H., Yuan G. (2014). Design and implementation of an optimized double closed-loop control system for MEMS vibratory gyroscope. IEEE Sens. J..

[7] Tsai N., Sue C. (2010). Experimental analysis and characterization of electrostatic-drive tri-axis micro-gyroscope. Sens. Actuators A.

[8] Liu H., Meng R. (2013). Self-oscillation loop design and measurement for an MEMS resonant accelerometer. Int. J. Adapt. Control Signal Processing.

[9] Su Y., Xu P., Han G., Si C., Ning J., Yang F. (2020). The Characteristics and Locking Process of Nonlinear MEMS Gyroscopes. Micromachines.

[10] Ulrike N., Michael C., Jan E.M., Peter D.S. Nonlinear dynamical system model for drive mode amplitude instabilities in MEMS gyroscopes. Proceedings of the 2019 IEEE International Symposium on Inertial Sensors and Systems (INERTIAL).

[11] Luo S., Ma H., Li F., Ouakad H.M. (2022). Dynamical analysis and chaos control of MEMS resonators by using the analog circuit. Nonlinear Dyn..

[12] Ehsan M., Aghil Y., Hossein N., Farid T. (2015). Study of nonlinear dynamics and chaos in MEMS/NEMS resonators. Commun. Nonlinear Sci. Numer. Simul..

[13] Yin Y., Fang Z., Han F., Yan B., Dong J., Wu Q. (2017). Design and test of a micromachined resonant accelerometer with high scale factor and low noise. Sens. Actuators A.

[14] Wang Y., Zhang J., Yao Z. (2018). A MEMS resonant accelerometer with high performance of temperature based on electrostatic spring softening and continuous ring-down technique. IEEE Sens. J..

[15] Marco B., Giorgio M., Christian P., Andrea D., Carlo V. (2021). On amplitude-gain-control optimization for Lissajous frequency modulated MEMS gyroscopes. IEEE Sens..

[16] Tomas M., Peter G.S., Farbod A., Murali K.G. (2020). Method to determine the closed-loop precision of resonant sensors from open-loop measurements. IEEE Sens. J..

[17] Miller J.M.L., Shin D.D., Kwon H.K., Shaw S.W., Kenny T.W. (2019). Phase control of self-excited parametric resonators. Phys. Rev. Appl..

[18] Higuchi E., Yabuno H., Yamamoto Y., Matsumoto S. (2022). Experimental amplitude and frequency control of a self-excited microcantilever by linear and nonlinear feedback. J. Micromech. Microeng..

[19] An L., Yabuno H. (2022). Self-excited oscillation produced by a phase shift: Linear and nonlinear instabilities. Nonlinear Dyn..

[20] Hossein S.H., Amir D.M. (2012). Resonance tracking of nonlinear MEMS resonators. IEEE/ASME Trans. Mechatron..

[21] Hutomo S.W., Zhang Q., Stephan M., Wang A., Peiner E. (2014). A phase locked loop frequency tracking system for portable microelectromechanical piezoresistive cantilever mass sensors. Microsyst. Technol..

[22] XU J., Li H., Wang X., Liu D., Feng L. (2020). Stability design of resonance frequency tracking system for sensing resonator. IEEE Sens. J..

[23] Antonio D., Zanette D.H., López D. (2012). Frequency stabilization in nonlinear micromechanical oscillators. Nat. Commun..

[24] Li H., Xu J., Wang X., Liu D., Feng L. (2020). High-Bandwidth Tracking Method of Resonant Frequency for Sensing Resonators. J. Lightwave Technol..

[25] Indeitsev D.A., Belyaev Y.V., Lukin A.V., Popov I.A. (2021). Nonlinear dynamics of MEMS resonator in PLL-AGC self-oscillation loop. Nonlinear Dyn..

[26] Lukin A.V. Method for determining the stability regions of stationary oscillations of a nonlinear MEMS resonator under the action of phase-locked-loop and automatic gain control systems. Proceedings of the 28th Saint Petersburg International Conference on Integrated Navigation Systems (ICINS).

[27] Wang X., Zheng X., Wu H., Pang Q., Shen Y., Ma Z., Jin Z. A Micromechanical Mode-Matched Gyroscope Using Stiffness Nonlinearity and Electrostatic Tuning. Proceedings of the 2021 IEEE 34th International Conference on Micro Electro Mechanical Systems (MEMS).

